# Neural Epidermal Growth Factor-Like Like Protein 2 Is Expressed in Human Oligodendroglial Cell Types

**DOI:** 10.3389/fcell.2022.803061

**Published:** 2022-02-21

**Authors:** Mohammed R. Shaker, Amna Kahtan, Renuka Prasad, Ju-Hyun Lee, Giovanni Pietrogrande, Hannah C. Leeson, Woong Sun, Ernst J. Wolvetang, Andrii Slonchak

**Affiliations:** ^1^ Australian Institute for Bioengineering and Nanotechnology, The University of Queensland, Brisbane, QLD, Australia; ^2^ St Cloud Technical & Community College, St Cloud, MN, United States; ^3^ Department of Anatomy, Brain Korea 21 Plus Program for Biomedical Science, Korea University College of Medicine, Seoul, South Korea; ^4^ School of Chemistry and Molecular Biosciences, The University of Queensland, Brisbane, QLD, Australia

**Keywords:** brain organoid, NELL2, neural stem cells, oligodendrocyte, artificial intelligence

## Abstract

Neural epidermal growth factor-like like 2 (NELL2) is a cytoplasmic and secreted glycosylated protein with six epidermal growth factor-like domains. In animal models, NELL2 is predominantly expressed in neural tissues where it regulates neuronal differentiation, polarization, and axon guidance, but little is known about the role of NELL2 in human brain development. In this study, we show that rostral neural stem cells (rNSC) derived from human-induced pluripotent stem cell (hiPSC) exhibit particularly strong *NELL2* expression and that NELL2 protein is enriched at the apical side of neural rosettes in hiPSC-derived brain organoids. Following differentiation of human rostral NSC into neurons, NELL2 remains robustly expressed but changes its subcellular localization from >20 small cytoplasmic foci in NSC to one–five large peri-nuclear puncta per neuron. Unexpectedly, we discovered that in human brain organoids, NELL2 is readily detectable in the oligodendroglia and that the number of NELL2 puncta increases as oligodendrocytes mature. Artificial intelligence-based machine learning further predicts a strong association of NELL2 with multiple human white matter diseases, suggesting that NELL2 may possess yet unexplored roles in regulating oligodendrogenesis and/or myelination during human cortical development and maturation.

## Introduction

The neural tube is the precursor of the brain and spinal cord and is derived from embryonic neural stem cells (NSCs) (Pai et al., 2012). During early central nervous system (CNS) development, rostral and caudal NSCs are derived from distinct lineages, namely, neuroectoderm (NEct) and neuromesodermal progenitors (NMPs), respectively (Shaker M. R. et al., 2021; Shaker et al., 2021c; Hudson and Yasuo, 2021). These two distinct populations also differ in proliferation rates, differentiation potential, and ECM adhesion (Shaker et al., 2015; Shaker M. R. et al., 2020). NSCs are generally defined as self-renewing multipotent cells that can differentiate into neurons, astrocytes, and oligodendrocytes (Lee et al., 2020a). NSCs can be found across the brain and spinal neural tube during development and are maintained to adulthood to meet the demand of adult neurogenesis and gliogenesis (Sabelström et al., 2014; Kim et al., 2015; Rusznák et al., 2016). Therefore, defining the molecular processes that regulate NSCs is central to the development of future regenerative therapeutic strategies for the CNS.

Neural epidermal growth factor-like like 2 (NELL2) is a protein kinase C-binding protein that is predominately expressed in the CNS of humans, mammals, and amphibians (Watanabe et al., 1996). Structurally, Nell2 is a cytoplasmic glycosylated protein with six epidermal growth factor-like domains (cNell2) and five von Willebrand factor (v.WF) C domains. In animal models, Nell2 has been shown to have multiple roles in regulating neuronal proliferation, survival, differentiation, polarization, as well as axon guidance and synaptic functions (Aihara et al., 2003; Nelson et al., 2004; Jeong et al., 2008). More specifically in rodents, Nell2 expression gradually increases during development to adulthood and is expressed in the tania tecta, piriform cortex, hippocampus, cerebellar cortex, and dentate gyrus (Ha et al., 2013). An increasing body of evidence suggests a pivotal role of Nell2 in CNS development. For instance, it binds to and promotes oligomerization of Robo3 *via* the EGF domains (Jaworski et al., 2015), resulting in the repulsion of commissural axons in the midline (Pak et al., 2020). Nell2 also controls retinal ganglion cell wiring in rodents (Pak et al., 2020), and in the developing spinal cord, it is also widely expressed in the ventral horn of the motor column and in the dorsal root ganglia (Jaworski et al., 2015). In addition to these roles, secreted isoforms of Nell2 (sNell2) can promote neuronal survival (Aihara et al., 2003), and ectopic expression of sNell2 in neuronal progenitor HiB5 cells promotes their differentiation (Kim et al., 2010). To date, little is known about the expression pattern and the roles of NELL2 in human CNS development.

Our data revealed a two-fold higher expression of *NELL2* expression in rostral versus caudal NSCs in both human and murine cells, which was confirmed for both the secreted and cytosolic forms of NELL2. To investigate the role of NELL2 in CNS development, we generated human brain organoids and detected abundant NELL2 expression at the apical side of neural rosettes, as well as in neuronal progenitors and post-mitotic neurons, but not in astrocytes. Intriguingly, we also discovered that, in contrast to what has been reported in animal models, NELL2 is robustly expressed in the oligodendrocyte lineage. This prompted us to investigate the relevance of NELL2 roles in oligodendrocytes *via* AI-based analysis, revealing a previously unknown link between NELL2 and white matter diseases.

## Results

### Gene Expression Comparisons of Mouse CNS and mES Cell-Derived Neural Stem Cells Identify Nell2 as a Predominantly Rostral Neural Stem Cell-Expressed Gene

**FIGURE 1:**
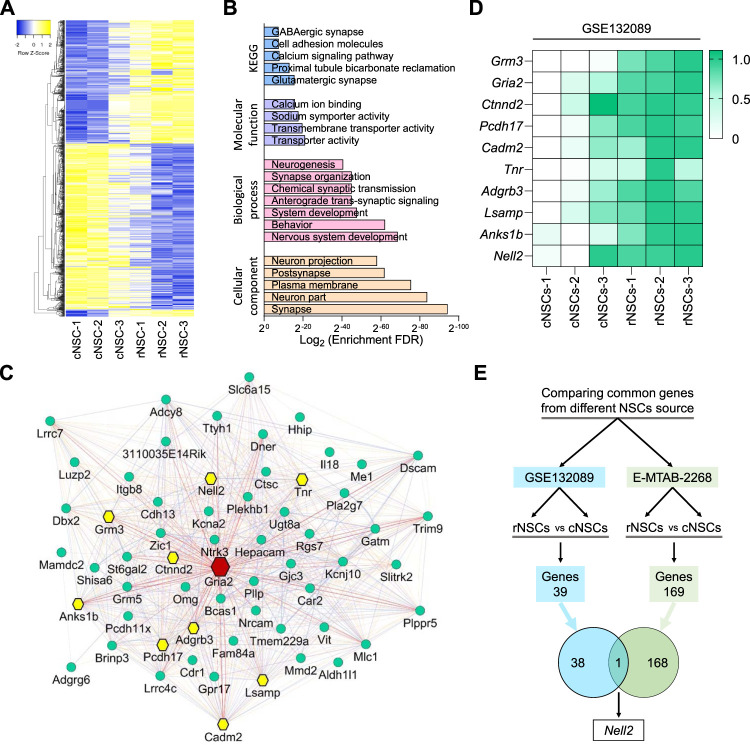
Microarray and network hub gene analysis of differentially expressed genes in the developing mouse central nervous system. **(A)** Heatmaps of differentially expressed genes in rostral compared to the caudal neural stem cells (NSCs). rNSCs, rostral NSCs; cNSCs, caudal NSCs. **(B)** Bar graphs showing KEGG (blue), molecular function (purple), biological process (pink), and cellular component (light orange) enrichment analysis of upregulated genes in rostral NSCs compared to caudal NSCs. **(C)** Network plot of top 20% genes enriched in rostral NSCs; the node in red is the main hub gene with degree = 68, nodes in yellow are hub genes with degree of ≥50, and nodes in green are genes of a degree ≤50. Interactive network is generated using GeneMANIA plugin in Cytoscape, and interactions between genes are generated based on co-expression, co-localization, physical interactions, shared protein domains, and genetic interactions. **(D)** Heatmap representing the expression of microarray data of mouse hub genes in caudal and rostral NSCs from a published dataset (GSE132089) (Shaker M. R. et al., 2020). Relative expression data was further normalized between 0 (lowest) and 1 (highest) expression value of individual genes across datasets. Normalized expression values were color-coded with green values indicating upregulation and white values indicating downregulation. rNSCs, rostral NSCs; cNSCs, caudal NSCs. **(E)** Venn diagram showing the overlap of upregulated gene (*Nell2*) in NSCs derived from mouse ESCs *in vitro* [green, E-MTAB-2268 (Gouti et al., 2014)] and in mouse *in vivo* primary embryonic NSCs [blue, GSE132089 (Shaker M. R. et al., 2020)].

In order to gain insight into the gene expression profiles of NSCs, we utilized a published dataset (GSE132089) (Shaker M. R. et al., 2020) and compared gene expression profiles of mouse NSCs derived from different axes along the embryonic neural tube. This revealed distinct gene expression signatures of NSC along the anteroposterior axis of the developing CNS (Figure 1A) and identified 267 genes that are enriched in rostral NSCs (rNSCs) compared to caudal NSCs (cNSCs) (Supplementary Table S1). Gene Ontology enrichment analysis indicated that these 267 rNSC genes are enriched for genes involved in synapse, nervous system development, transport activity, and glutamatergic synapse formation (Figure 1B). To gain insight into the up-regulated genes, we next selected the top 20% of genes enriched in rNSCs (Supplementary Table S1) and generated an interactive network based on gene co-expression, co-localization, and physical interaction (Figure 1C) and further analyzed the node degree of each gene in this network with a Cytoscape GeneMANIA plugin. Since highly connected hub genes are more likely to play an essential role in controlling biological processes compared to lowly connected genes (Langfelder et al., 2013), genes with the highest node degree were treated as a main hub gene in this network. This analysis revealed that the *Gria2* gene was a central hub gene with a node degree score of 60 (Figure 1C, red node), followed by secondary hub genes, which included *Nell2* with a node score of 51 as well as *Tnr*, *Grm3*, *Ctnnd2*, *Anks1b*, *Adgrb3*, *Pcdh17*, *Lsamp*, and *Cadm2* (Figure 1C, yellow nodes). Heatmap analysis of these 10 hub genes in mouse NSCs confirmed the significant enrichment in rNSCs compared to the cNSCs (Figure 1D), as expected. We next compared the mouse embryo-derived NSC (GSE132089) dataset with the gene expression profile of mES cell-derived rostral and caudal NSCs (E-MTAB-2268) (Gouti et al., 2014) (Figure 1E). Interestingly, we identified only one gene common between these two rNSC populations, namely, *Nell2*. Collectively, our gene expression and network analysis thus revealed that *Nell2* is one of the 10 hub genes with the potential to regulate the function of rNSCs during embryonic development and identified *Nell2* as a standout gene that is expressed in rNSC derived from mouse embryos and mES cells.

### Cytosolic and Secreted NELL2 Most Strongly Expressed in Rostral Neural Stem Cells

**FIGURE 2:**
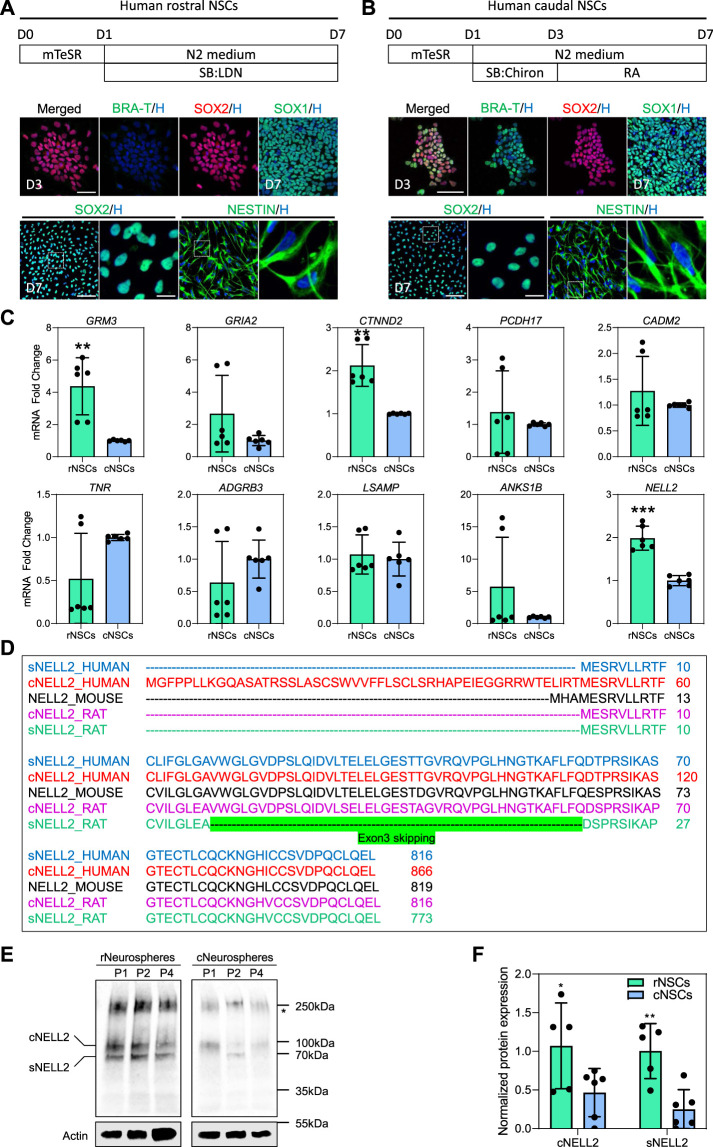
Expression of murine hub genes and analysis of cytosolic and secreted isoforms of human NELL2 in human-induced pluripotent stem cell (iPSC)-derived neural stem cells. **(A)** Schematic diagram of experimental procedure for generation human rostral NSCs, followed by immunohistochemistry confirmation. WTC iPSCs were differentiated towards neuroectodermal progenitors and further differentiated into rostral NSCs. Below images represent staining of neuroectodermal progenitors with BRA-T (green) and SOX2 (red) at day 3 of differentiation; rostral NSCs are stained with SOX1 (green), SOX2 (green), and NESTIN (green); all nuclei were counterstained with Hoechst 33342 (blue). Scale bar of upper images is 30 μm; scale bar of bottom images is 50 μm; zoomed images’ scale bar is 10 µm. Dotted box indicates the magnified region. D, day. **(B)** Schematic diagram of experimental procedure for generation human caudal NSCs. WTC iPSCs were differentiated towards neuromesodermal progenitors and then differentiated further into caudal NSCs. Below images represent staining of neuromesodermal progenitors with BRA-T (green) and SOX2 (red) at day 3 of differentiation; caudal NSCs are stained with SOX1 (green), SOX2 (green), and NESTIN (green); all nuclei were counterstained with Hoechst 33342 (blue). Scale bar of upper images is 30 μm; scale bar of bottom images is 50 μm; zoomed images’ scale bar is 10 µm. Dotted box indicates the magnified area. D, day. **(C)** qRT-PCR of 10 identified murine hub genes (*GRM3*, *GRIA2*, *CTNND2*, *PCDH17*, *CADM2*, *TNR*, *ADGRB3*, *LSAMP*, *ANKS1B*, and *NELL2*) in human rostral and caudal NSCs. All values were normalized to GAPDH levels of their respective samples and expressed relative to caudal NSC values to obtain the fold change. Data are shown as mean ± standard deviation; number of independent experiments = 6. **p < 0.01, ***p < 0.001 *via* Student’s *t*-test. rNSCs, rostral NSCs; cNSCs, caudal NSCs. **(D)** Amino acid sequence of cytosolic and secreted NELL2 in humans, mice, and rats. Green highlights the exon 3 skipping in secreted rat Nell2. cNELL2, cytosolic NELL2; sNELL2, secreted NELL2. Full-length sequences can be found in Supplementary Table S5. **(E)** Representative western blots showing the protein levels of cytosolic and secreted forms of NELL2 in human WTC iPSC-derived rostral and caudal neurospheres at passages 1, 2, and 4. Actin was used for normalization. Star indicates the unknown bands of NELL2. All blots derive from the same experiment and were processed in parallel. P, passage; rNSCs, rostral NSCs; cNSCs, caudal NSCs; cNELL2, cytosolic NELL2; sNELL2, secreted NELL2. **(F)** Bar graphs showing the quantification of cytosolic and secreted forms of NELL2 levels obtained from panel **(E)**. Data are shown as mean ± standard error mean. The number of independent experiments = 5; *p < 0.05; **p < 0.01 *via* Student’s *t*-test. rNSCs, rostral NSCs; cNSCs, caudal NSCs; cNELL2, cytosolic NELL2; sNELL2, secreted NELL2.

Since little is known about the 10 murine NSC hub gene expression in NSC of the early developing human CNS, we next investigated their expression in rNSCs and cNSCs derived from human-induced pluripotent stem cells (hiPSCs). To this end, human WTC iPSCs (Kreitzer et al., 2013) were differentiated into NEct or NMP using our published protocols (Shaker M. R. et al., 2020; Shaker et al., 2021a) to generate populations of rNSCs and cNSCs, respectively (Figure 2A, B). As expected, human rNSCs and cNSCs both expressed the pan-NSC markers SOX1, SOX2, and NESTIN (Figure 2A, B), while only NMPs expressed BRA-T, confirming their caudal identity. qRT-PCR analysis of the 10 murine hub genes identified in Figure 1C in human rNSCs next revealed that human rNSCs expressed about twice as much *NELL2* as compared to cNSCs (Figure 2C). In addition, we also found *GRM3* and *CTNND2* were significantly enriched in human rNSCs as compared to cNSCs (Figure 2C). These data therefore indicate that a subset of mouse rNSC-enriched genes is conserved in *in vitro*-derived human rNSCs, supporting the notion that *NELL2* may also play important roles in human rNSCs during development.

Nell2 is expressed as both a secreted and a cytosolic isoform in murine tissues (Hwang et al., 2007). Hence, we first compared the amino acid sequence conservation of Nell2 between rodents, chicken, and human. As shown in Figure 2D, the secreted form of Nell2 is highly conserved between species with the exception of an exon 3-skipping event that generates a secreted form of Nell2 in the rat that is not found in humans. In contrast, the cytosolic form of human NELL2 possesses a unique human-specific sequence in the first exon that is not observed in other species. To further confirm this, we utilized an antibody that detects both isoforms and conducted a western blot analysis on human iPSCs-neurospheres derived from rNSCs and cNSCs at different passages. This western blot confirmed the expression of the secreted and cytosolic isoforms of NELL2 (Figure 2E), and in agreement with mRNA expression analysis, the protein level of the secreted and cytosolic NELL2 protein was two-fold higher in rostral neurospheres as compared to caudal neurospheres across different passages (Figure 2F).

### Cellular Distribution of NELL2 in Neural Cells of the Developing Human Fetal CNS

**FIGURE 3:**
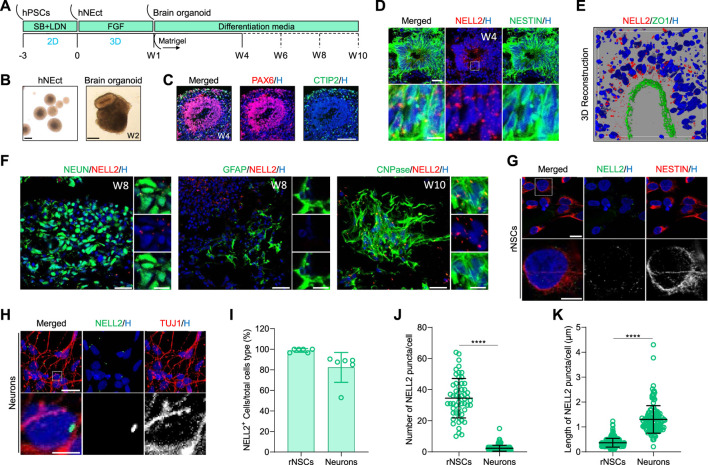
Analysis of NELL2 expression patterns in neural cells of human brain organoids. **(A)** Schematic representation of the strategy used to generate human cortical brain organoids from WTC iPSCs. hNEct, human neuroectodermal; W, week; D, day. **(B)** Representative images of human neuroectodermal spheroids after 4 days of FGF treatments and cortical brain organoids at 2 weeks of differentiation. Scale bar = 200 μm. **(C)** Representative images of sections of 4-week-old brain organoids derived from human WTC iPSCs, immunostained for ventricle zone PAX6 (red) and cortical plate CTIP2 (green). All sections were counterstained with Hoechst 33342 (blue). Scale bar = 120 μm. **(D)** Representative images of 4-week-old cortical brain organoid sections immunostained for NESTIN (green) and NELL2 (red). All sections were counterstained with Hoechst 33342 (blue). Scale bar = 30 μm; zoomed images’ scale bar = 10 μm. Dotted box indicates the magnified region. **(E)** Representative image of 3D reconstruction immunostained sectioned neural rosettes in organoids at 4 weeks old for ZO1 (green) and NELL2 (red). The section was counterstained with Hoechst 33342 (blue). **(F)** Representative images of cortical brain organoid sections immunostained for NEUN (green), GFAP (green), CNPase (green), and NELL2 (red). All sections were counterstained with Hoechst 33342 (blue). Scale bar = 30 μm; zoomed images’ scale bar = 10 μm. **(G)** Representative images of rostral neural stem cells derived from human WTC iPSCs. Rostral neural stem cells stained with NESTIN (red) and NELL2 (green). Scale bar = 16 μm. All nuclei were counterstained with Hoechst 33342 (blue). Dotted box indicates the magnified area. **(H)** Representative images of neurons derived from human WTC iPSCs. Rostral neural stem cells stained with TUJ1 (red) and NELL2 (green). Scale bar = 20 μm; zoomed images’ scale bar = 5 μm. All nuclei were counterstained with Hoechst 33342 (blue). Dotted box indicates the magnified area. **(I)** Percentage of NELL2^+^ cells relative to the total number of NESTIN^+^ and TUJ1^+^ cells in rNSCs and neurons, respectively. Data are presented as mean ± standard deviation. Number of independent experiments = 6. **(J)** Quantification of the number of NELL2 puncta per cells in rostral neural stem cells and neurons of the data presented in panels **(G,H)**. At least 223 cells were counted per three biological replicates. Data are presented as the mean ± standard deviation. ****p < 0.0001 *via* Student’s *t*-test. rNSCs, rostral NSCs. **(K)** Measurement of the length of NELL2 puncta per cell in rostral neural stem cells and neurons of data presented in **(G,H)**. At least 223 cells were counted per three biological replicates. Data are presented as the mean ± standard deviation. ****p < 0.0001 *via* Student’s *t*-test. rNSCs, rostral NSCs.

Previous immunofluorescence studies in rat and chicken CNS tissues reported that Nell2 was exclusively present in brain neurons (Nelson et al., 2004; Ha et al., 2008; Kim et al., 2020), but little is known about its expression in the developing human brain. To address this knowledge gap, we investigated NELL2 expression in human brain organoids generated from human ESC and iPSCs because brain organoids mimic the cellular make-up of early developing human brain, including NSCs, neurons, oligodendroglia, and astrocytes (Shou et al., 2020). The cortical brain organoids we generated from human NEct cells with our previously developed protocol (Shaker et al., 2021a) exhibited the layered architecture of the developing cortex with multiple neurogenic zones (Figure 3A, B). Immunostaining of 4-week-old brain organoid sections with PAX6 and CTIP2 antibodies confirmed the successful generation of ventricular zone and cortical plate (cortical layer V), respectively (Figure 3C). In brain organoids, NSCs can be identified based on NESTIN expression in neural rosettes that are clearly evident in the brightfield images (Figure 3B). Analysis of NELL2 expression in the 4-week-old brain organoids revealed widespread expression of NELL2 in ∼90% of NSCs (Figure 3D). A particularly high expression of NELL2 was detected in NSCs that were apically located within the neural rosettes compared to cells positioned close to the basal level, and this was confirmed by confocal imaging of human neural rosettes double labelled with NelL2 and ZO1 antibodies (Figure 3E). These data are consistent with previous studies in human cell lines and rats that indicated Nell2 modulates cell proliferation and growth (Kim et al., 2020; Liu et al., 2021). Since human iPSC-derived cortical brain organoids contain both mature neurons and glia, we next analyzed the expression of NELL2 in these cell types in 8- and 10-week-old brain organoids by co-staining with NEUN for neurons, GFAP for astrocytes, and CNPase for oligodendrocytes. We found that 94.5% of neurons exhibited one–two large condensed elongated NELL2 puncta (Figure 3F), while GFAP-expressing cells were largely devoid of NELL2 immunoreactivity (Figure 3F). Surprisingly, we discovered that the vast majority (97.3%) of oligodendrocytes marked by CNPase exhibited a particularly high density of NELL2 puncta in 10-week-old brain organoids (Figure 3F). We noted that NELL2 is detected as multiple small puncta in NSCs compared to a few large and elongated puncta in neurons and oligodendrocytes. To further explore this, we generated iPSC-derived monolayer rNSCs and post-mitotic neurons. Immunocytochemistry detection of NELL2 protein again revealed the expression of NELL2 in almost all human rNSCs marked by NESTIN expression (Figure 3G–K). Quantification revealed 16–65 small puncta of NELL2 that were localized across the cell body of NESTIN-expressing cells (Figure 3J). Subsequent differentiation of rNSCs into day 14 neurons revealed that the number of puncta per TUJ1-expressing neuron was dramatically reduced to one–five puncta per neuron (Figure 3J) and that these NELL2 puncta were significantly larger and elongated as compared to rNSCs (Figure 3K). Collectively, these data reveal that NELL2 is expressed in both human iPSC-derived NSCs and neurons, but that the number and shape of the cytosolic NELL2 puncta change as cells differentiate from NSCs to neurons.

### NELL2 Expression During Human Oligodendrocyte Generation and Maturation

**FIGURE 4:**
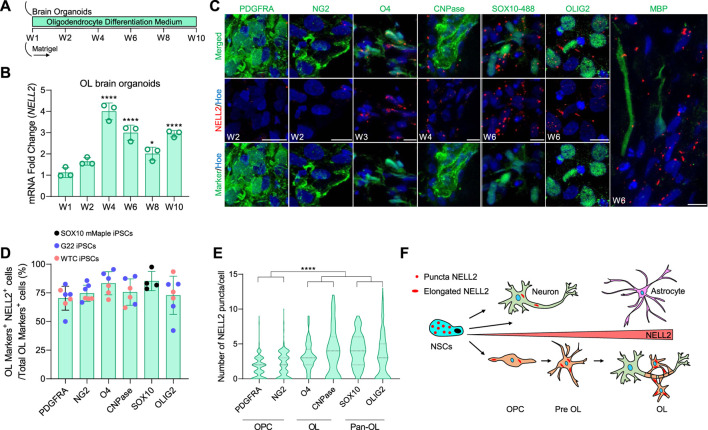
Detailed analysis of NELL2 expression patterns across oligodendroglial cells in human OL brain organoids. **(A)** Schematic representation of the strategy used to generate human oligodendrocyte brain organoids from WTC iPSCs, G22 iPSCs, and SOX10 mMaple iPSCs. W, week. **(B)** qRT-PCR of *NELL2* mRNA in human oligodendrocyte brain organoids. All values were normalized to GAPDH levels of their respective samples over week 1 to week 10 and expressed relative to week 1 to obtain the fold change. Data are shown as mean ± standard deviation; number of independent experiments = 3. *p < 0.05; ****p < 0.001 *via* one-way ANOVA. W, week; OL, oligodendrocyte. **(C)** Representative images of 2- and 3-week-old oligodendrocyte brain organoid sections immunostained for PDGFRA (green), NG2 (green), O4 (green), CNPase (green), OLIG2 (green), MBP (green), and NELL2 (red). For SOX10–488, week 6 oligodendrocyte brain organoids are derived from SOX10 mMaple iPSCs and stained with NELL2 (red). All sections were counterstained with Hoechst 33342 (blue). Scale bar = 10 μm. **(D)** Percentage of NELL2+ cells relative to the total number of PDGFRA+, NG2+, O4+, CNPase+, SOX10+, and OLIG2+ cells in oligodendrocyte brain organoids at week 2, week 3, week 4, and week 6. Data are presented as mean ± standard deviation. The minimum number of independent experiments = 4. Number of examined organoids = 36. **(E)** Quantification of the number of NELL2 puncta per cells in PDGFRA+, NG2+, O4+, cnpase+, SOX10+, and OLIG2+ cells in oligodendrocytes brain organoids at week 2, week 3, week 4, and week 6; data presented in panel **(C)**. At least 400 cells were counted per minimum three biological replicates. Data are presented as the mean ± standard deviation. ****p < 0.0001 *via* one-way ANOVA. **(F)** Schematic diagram of puncta and elongated form of NELL2 distribution across human embryonic neural cells.

To further explore the previously not reported expression of NELL2 in oligodendrocytes, we took advantage of a rapid and efficient protocol recently developed in our laboratory that fosters the maturation and enrichment of oligodendrocytes in brain organoids (OL brain organoids) (Shaker M. R. et al., 2021) (Figure 4A). Quantification of *NELL2* mRNA expression by qPCR over the time course of OL brain organoid maturation revealed a significant upregulation of *NELL2* mRNA during the oligodendrocyte maturation period (at 4–10 weeks) (Figure 4B). A detailed analysis of NELL2 expression during oligodendrogenesis in the OL brain organoids at different stages of *in vitro* differentiation again revealed that the majority of oligodendroglia express NELL2 (Figure 4C, D). Quantification of NELL2 puncta further revealed a gradual and significant increase in the expression of NELL2 in maturing O4+ oligodendrocytes at week 3, CNPase+ oligodendrocytes at week 4, and SOX10+ cells at week 6 compared to PDGFRA and NG2-expressing oligodendrocyte progenitors at week 2 (Figure 4C, E). We further detected an abundance of NELL2 expression in cells expressing the pan-oligodendrocyte marker OLIG2 as compared to PDGFRA and NG2-expressing progenitors (Figure 4E). To further substantiate that cNELL2 was expressed by oligodendrocytes, we dissociated the 12-week-old brain organoids into single cells to remove secreted proteins and MACS-sorted O4^+^ oligodendrocytes from these single-cell populations (Supplementary Figure S1A). Western blot analysis of these enriched cell populations revealed a strong expression of 100-kDa NELL2 isoforms in O4^+^ cells and in the remaining brain organoid cells that express SOX10 and GFAP, respectively (Supplementary Figure S1B).

### Transcriptome Analyses of NELL2 in Developing Human Brain and Spinal Cord

**FIGURE 5:**
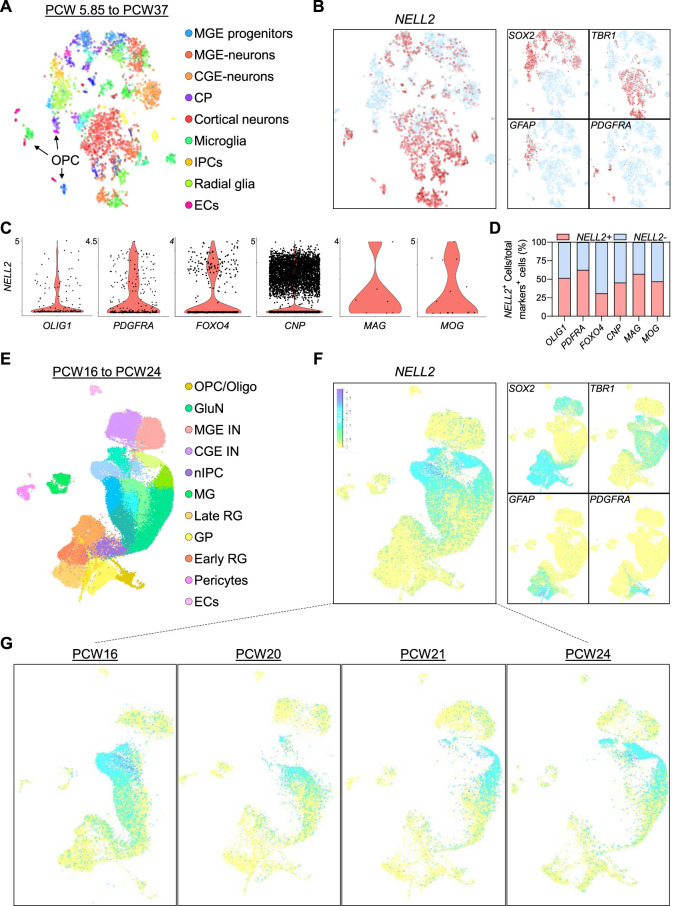
Transcriptome profiling of NELL2 in human fetal brain cells. **(A)** Two-dimensional tSNE plot of 4,261 cells colored by cluster of human fetal neural cells adopted from bit.ly/cortexSingleCell (Nowakowski et al., 2017). **(B)** The distribution of *NELL2* expression across the fetal brain neural cell clusters identified in panel **(A)**. **(C)** Violin plots showing the NELL2 expression across oligodendroglial cells markers including OLIG1, PDGFRA, FOXO4, CNP, MAG, and MOG across all clusters. The *x*-axis is the expression level. **(D)** Quantification of the percentage of NELL2+ cells relative to the total number of OLIG1+, PDGFRA+, FOXO4+, CNP+, MAG+, and MOG+ cells in oligodendrocyte human fetal brain. **(E)** UMAP plot of cells colored by cluster of human fetal neural cells adopted from bit.ly/cortexSingleCell (Trevino et al., 2021). RG, radial glia; OPC/Oligo, oligodendrocyte progenitor cell/oligodendrocyte; nIPC, neuronal intermediate progenitor cell; GluN, glutamatergic neuron; CGE IN, caudal ganglionic eminence interneuron; MGE IN, medial ganglionic eminence interneuron; EC, endothelial cell; MG, microglia. **(F)** The distribution of *NELL2* expression across the fetal brain neural cell (Sox2+, TBR1+, GFAP+, and PDGFRA+ cells) clusters identified in panel **(E)**. **(G)** The distribution of *NELL2* expression across the fetal brain neural cells at different stages of brain development.

Since our data indicate striking differences between human NELL2 and murine Nell2 expression, and to independently validate our findings, we next examined *NELL2* mRNA expression in published single-cell expression datasets derived from the developing human fetal brain (Nowakowski et al., 2017; Trevino et al., 2021) and spinal cord (Rayon et al., 2021). Clustering of single-cell analysis identified cell clusters corresponding to astrocytes, radial glia/NSCs, oligodendrocyte progenitors, mature neurons, microglia, as well as progenitors and neurons of the medial ganglionic eminence (MGE) (Figure 5A, E, Supplementary Figures S2, S3C). In agreement with our brain organoid data, *NELL2* expression was detected in subpopulations of NSCs, was abundant in mature cortical neurons, was minimally expressed in astrocytes, and was readily detectable in oligodendroglial cells (Figure 5B, F). Next, we examined whether the distribution of *NELL2* in human fetal brain oligodendroglial cells exhibits similar expression patterns compared to the oligodendroglial cells derived from the OL brain organoids. The quantification of the percentage of oligodendroglial cells expressing *NELL2* revealed an average of 49% of oligodendroglial cells that were *NELL2* ^+^ (Figure 5C, D) compared to 80% oligodendroglial cells that were NELL2^+^ in human OL brain organoids (Figure 4D). Interestingly, the expression of *NELL2* increased in primary oligodendroglial cells during fetal brain development (Figure 5G), in agreement with our OL brain organoid data. Of note, in these 3-month-old human embryonic brain cells, *NELL2* is also found in cells of the choroid plexus (CP) and MGE (Figures 5B,F).

**FIGURE 6:**
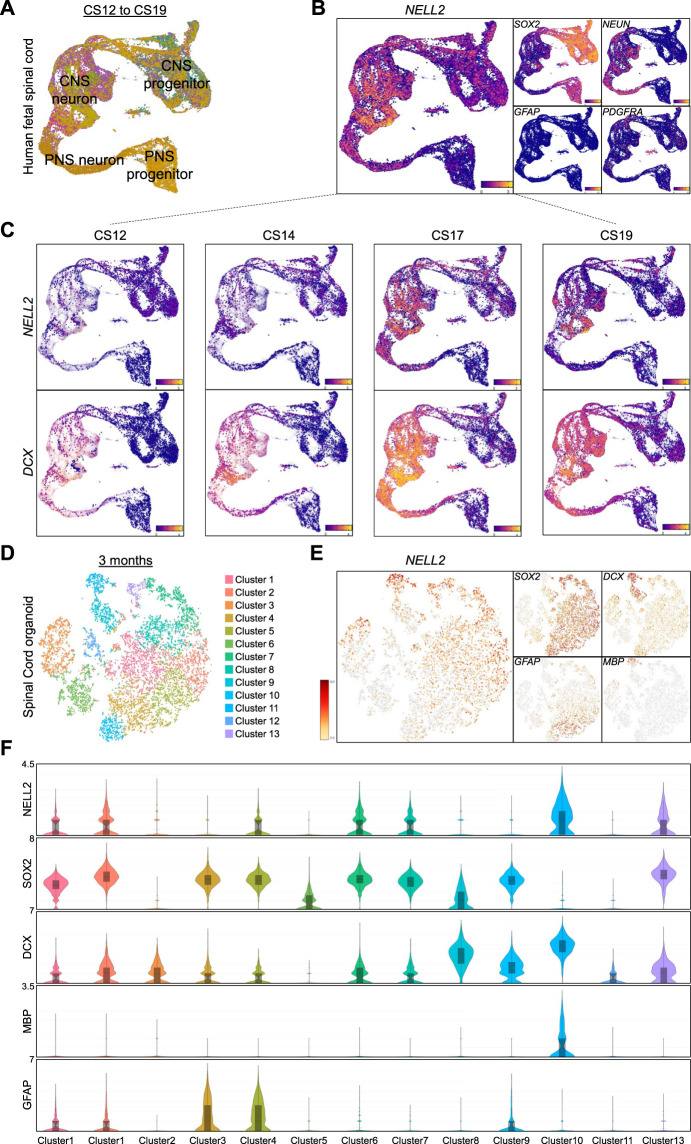
Transcriptome profiling of NELL2 in human fetal spinal cord cells. **(A)** UMAP plot of 43,485 cells colored by gene module score of clusters of CNS progenitor, CNS neurons, PNS progenitors, and PNS neurons adopted from human fetal neural cells adopted from https://shiny.crick.ac.uk/scviewer/neuraltube/ (Rayon et al., 2021). **(B)** Feature plots of markers of *NELL2*, neural progenitors (*SOX2*), neurons (*NEUN*), astrocytes (*GFAP*), and oligodendrocyte progenitors (*PDGFRA*). **(C)** The distribution of *NELL2* and *DCX* expression across the fetal spinal cord cells at different stages of development. **(D)** Two-dimensional tSNE of 10,361 cells from 3-month-old hSCOs *via* single-cell RNA sequencing identified by clustering specific transcription factors of dorsal and ventral spinal cord cells. **(E)** Gene expression profiles of *NELL2*, neural progenitors (*SOX2*), neuronal-specified progenitors (*DCX*), astrocytes (*GFAP*), and mature oligodendrocyte (MBP). **(F)** Violin plots showing the NELL2, SOX2, DCX, GFAP, and MBP (as defined by scRNA-seq) across the 13 clusters. The *x*-axis is log2 expression.

In human fetal spinal cord cells, widespread *NELL2* expression was found in neuronal cells (Figure 6A, B) and in subsets of individual *SOX2* ^*+*^ CNS progenitors (Figure 6B). A comparison of *NELL2* and *DCX* expression in spinal cord-derived cells from CS12 to CS19 embryos shows *NELL2* expression commences in *DCX*-expressing CNS progenitors around CS17 (Figure 6C). To investigate whether this expression pattern was recapitulated *in vitro*, we generated human spinal cord organoids (hSCOs) as recently reported (Lee et al., 2020b) and carried out droplet-based single-cell RNA sequencing (scRNA-seq) of 3-month-old hSCOs with 10,361 cells. We performed a clustering analysis based on the representative transcription factors in spinal cord cells and found that these 3-month-old hSCOs were composed of 13 cell clusters (Figure 6D). We found a number of genes associated with dorsal and ventral subtypes of spinal cord cells (Supplementary Figure S3A). The detection of spinal cord *HOX* code genes further substantiated the spinal cord identity of these organoids (Supplementary Figure S3B). Markers of spinal cord NSCs, astrocytes, neuronal-restricted progenitors, and oligodendrocytes showed strong cluster-specific enrichments in these hSCOs (Figure 6E). *NELL2* expression was found to be associated with distinct cell clusters (Figure 6E, F) that varied in abundance from 1,468 to 257 cells (Supplementary Figure S3C). The expression levels of *SOX2*, *DCX*, *GFAP*, and *MBP* were used to define clusters of cNSCs, astrocytes, neuronal-restricted progenitors, and myelinating oligodendrocytes, respectively (Figure 6F). Consistently, all *SOX2* clusters that also showed an increase in *DCX* expression exhibited *NELL2* upregulation, except for cluster 5 that exhibited only upregulation of *SOX2* expression (Figure 6F). Similar to what we found in human brain cells, we again detected abundant *NELL2* in *MBP*-expressing oligodendroglial cells but not in GFAP-expressing astrocytic cell clusters (Figure 6F).

Collectively, our own brain and spinal cord organoid-derived data and single-cell transcriptome analyses, as well as published single-cell transcriptome data, substantiate that *NELL2* is expressed in human brain NSCs, neurons, and in oligodendrocytes as well as in spinal cord neurons and oligodendrocytes (Figure 4F), suggesting NELL2 may have as yet unexplored functions in human oligodendrocytes.

### Artificial Intelligence Analysis Predicts NELL2 Is Associated With White Matter Diseases

Artificial intelligence (AI)-based machine learning has accelerated translational medicine and drug discovery (Koohi-Moghadam et al., 2019; Vatansever et al., 2021). Given the fact that there are no studies of NELL2 in the developing human CNS and intrigued by the observation that human oligodendrocytes robustly express NELL2, we next utilized the AI-based screening tool iCLUE&ASK to explore whether NELL2 can be linked to diseases associated with oligodendrocyte maturation or function. Provocatively, our AI analysis linked NELL2 with multiple white matter diseases such as progressive demyelinating neuropathy with bilateral striatal necrosis, abnormal myelination, CNS demyelination, delayed myelination, and CNS hypomyelination (Supplementary Table S2), suggesting that NELL2 may be involved in oligodendrogenesis and/or maintaining myelin sheath integrity. Interestingly, the highest score of NELL2 prediction was shown for brain glioma. We also identified multiple neuronal-associated diseases such as Alzheimer’s disease, neurodevelopmental disorder, and sensorimotor neuropathy, consistent with our observation that NELL2 is abundant in post-mitotic neurons of the brain and spinal cord.

## Discussion

The rostral and caudal domains of the CNS are derived from completely distinct lineages, termed NEct and NMPs. NEct cells are the major progenitor population that give rise to the anterior neural tube, whereas NMP populations are located at the caudal end and form the caudal neural tube and progressively add new neural cells to build the elongating caudal neural tube (Shaker et al., 2021c). Because this tract of CNS development in vertebrates has only been discovered recently, relatively little is known in regulating the specific features of NSCs of rostral versus caudal origin.

To start to address this knowledge gap, we first compared mouse primary embryonic NSCs of caudal and rostral origin. This analysis allowed us to identify several hub genes of predicted relevance to CNS development. To further validate these genes, we performed an analogous process on rostral and caudal NSCs generated from mouse iPSCs *in vitro*. Intriguingly, this identified *Nell2* as the only hub gene that was common between these two datasets. We next used our previously established protocols to generate human rostral and caudal NSCs derived from iPSCs/ESCs *via* NEct and NMP lineages (Shaker M. R. et al., 2020). We demonstrate that 30% of mouse rostral hub genes are also expressed in human rNSCs, namely, *GRM3*, *CTNND2*, and *NELL2*. Among these three genes, *NELL2* was the only hub gene that was consistently detected across multiple published datasets, including in the human rNSCs generated in this study. Collectively, this indicates that *NELL2* is an important gene with functions that are likely conserved across mammalian species. Nell2 is a mammalian neural tissue-enriched EGF-like repeat domain-containing protein and contains multiple growth factor binding and signaling domains, suggesting complex multifactorial roles in neural cells (Kim et al., 2014). While it was previously reported that Nell2 is expressed in rat and chick neural tissues, Nell2 is not enriched in rodent NSCs. Here, we report for the first time that the *NELL2* gene is expressed in human rNSCs at two-fold higher levels than cNSCs derived from iPSCs. Human rNSCs in neural rosettes exhibited a high density of NELL2 protein that was located in multiple puncta-like structures in 2D and 3D human models. Neural rosettes are made of NSCs where apical cells marked by ZO1 maintain their proliferation and stemness to preserve the NSCs pool, and basal cells that exhibit tangential migration exit their cell cycle and differentiate into neurons (Lee et al., 2020b). Interestingly, we found a gradient of NELL2 expression along the apicobasal axis of the neural rosettes, with the highest level of NELL2 observed in apical cells compared to the basal cells. Our confocal 3D reconstruction imaging of the human rosettes strongly suggests Nell2 is intracellular (Figure 3E). Collectively, these data suggest that NELL2 may regulate the proliferation of rNSCs during development, in line with previous studies (Aihara et al., 2003; Nelson et al., 2004; Jeong et al., 2008) that demonstrated that NELL2 modulates cell proliferation and growth in human and rat cells (Kim et al., 2020; Liu et al., 2021). We certainly detected consistent differences in expression levels between rNSC and cNSC populations. Our data demonstrate that NELL2 is abundant in human rNSCs compared to cNSCs, but *NELL2* mRNA and protein expression persists in neuronal cell types derived from rNSCs, while cNSCs started to express *NELL2* as they upregulate the expression of *DCX* (restricted neuronal progenitor population) during human fetal spinal cord development. This is consistent with a previous study that demonstrated that Nell2 in chickens is also expressed by cells that are leaving the ventricular zone and coalesce in the mantle zone (Nelson et al., 2004), and forced expression of Nell2 in spinal neural progenitors located near the ventricular zone promotes this translocation to the mantle layer near the basal domain and their subsequent differentiation into neurons (Nelson et al., 2004). This identifies a marked difference in NELL2 expression pattern between the caudal and rostral NSCs and their neuronal progeny. It remains to be determined whether the function of NELL2 in these two CNS lineages is similar/different, although our data suggest distinct roles for NELL2 in rNSCs (promotion of proliferation) and cNSCs (promotion of differentiation). On the other hand, both rostral and caudal mature oligodendrocytes consistently express NELL2, suggesting a fundamental role of NELL2 in the maturation processes of oligodendrocytes in human CNS. In the future, it will be particularly interesting to carry out cell type-specific repression and overexpression studies of NELL2 in different cell types (such as rostral vs caudal NSC) in human iPSC-derived brain organoid models to further elucidate the precise cell type-specific roles of cytosolic and secreted NELL2 isoforms along the rostral–caudal axis of various stages of human CNS development.

Nell2 presence in mature neurons has been observed in numerous studies (Oyasu et al., 2000; Jeong et al., 2008). In mouse spinal cord, Nell2 is restricted to the motor and sensory neurons (Jaworski et al., 2015). While in rat brain, Nell2 is found in neurons populating the hippocampus and cerebellum (Oyasu et al., 2000; Jeong et al., 2008). In rats, Nell2 enhanced the survival of neurons from the hippocampus and cerebral cortex through mitogen-activated protein kinases (Aihara et al., 2003). In cultured hippocampal neurons, Nell2 promoted neuronal polarization and axon growth through the ERK signaling pathway (Kim et al., 2020). In agreement with these data, we found a significant expression of NELL2 in human cortical neurons that was concentrated in elongated and condensed puncta/vesicles. We also detected a significant enrichment of NELL2 in spinal cord neurons. Although Nell2 is predominantly expressed in the CNS during development, astrocytes in rodent models appeared to have no expression of Nell2 (Oyasu et al., 2000; Jeong et al., 2008). In line with these previous studies, we also failed to detect NELL2 in astrocytes derived from hiPSCs.

Nell2 protein has six EGF-like repeat domains that interact with the regulatory domain of protein kinase C in the cytosol (cNell2) (Hwang et al., 2007), but it also exists as secreted (sNell2) and thus can act as a diffusible ligand. The size of the sNell2 protein is smaller than the cNell2 in monkey (Kim et al., 2010), whereas rat sNell2 is larger than cNell2 (Hwang et al., 2007). Our data show that the human isoforms of NELL2 proteins examined in this study are similar to monkey Nell2 and confirmed that sNELL2 and cNELL2 exhibit molecular weights of 70 and 100 kDa, respectively. Our data further confirmed enrichment of cNELL2 in human neuronal cells (Supplementary Figure S1), suggesting potential involvement of cNELL2 in human cortical development and maturation. cNELL2 acts cell autonomously to promote differentiation of neural progenitors into neurons (Nelson et al., 2004), whereas sNell2 of neuronal origin can act in a paracrine fashion, among others stimulating the survival and proliferation of neighboring cells. Furthermore, murine sNell2 is widely expressed in neural tissues and reported to repel axons by binding to Robo3 receptor (Jaworski et al., 2015). Furthermore, cNell2 can bind to a protein kinase C isoform (i.e., PKCβ1) (Kuroda and Tanizawa, 1999), a finding that was subsequently confirmed in primary murine astrocytes, and there functions as an inhibitor of PKCβ1 (Lee et al., 2014). Since PKCβ1 is a known regulator of oligodendrocyte differentiation (Baron et al., 1998), it is tempting to speculate that the cytoplasmic form human NELL2 can similarly regulate oligodendrocyte differentiation, a role that is clearly absent in rodents. To unequivocally confirm that our *in vitro* expression data match *in vivo* NELL2 expression patterns, it will however be important to examine brain region-specific NELL2 expression in early human fetal brain sections at various stages of development with NELL-2 isoform-specific antibodies and/or *in situ* detection of mRNA. Our immunohistochemical analyses identified different patterns of NELL2 localization in human brain cells. In NSCs, NELL2 exhibited peri-nuclear localization in agreement with earlier reports of localized NELL2 protein expression in the rough ER (Hwang et al., 2007). In addition, our histological analysis showed that NELL2 is localized in condensed and longer puncta in mature neurons. The nature of these NELL2 puncta remains unclear at present, but we speculate that in human NSCs, these may represent vesicle-bound NELL2 destined for secretion. Given that NELL2 is glycosylated and has both cytoplasmic and extracellular roles, it is however also possible that the perinuclear punctate staining we observed with NELL2 antibodies in neuronal stem cells and their differentiated progeny mark specialized ER–Golgi contact sites that are known to be involved in sorting proteins to these two locations (Omari et al., 2020; Shomron et al., 2021). It remains to be determined whether a switch in subcellular localization, size, and aspect of the NELL2 puncta as cells differentiate from NSCs into neurons is related to different functions of the cytoplasmic versus *trans*-membrane or secreted forms of NELL2 in these cell types.

Perhaps most significantly, our extensive cellular and transcriptome analyses revealed that human oligodendroglia robustly express NELL2, whereas this cell type does not appear to express Nell2 in murine models. We demonstrate that hiPSC-derived oligodendrocytes are significantly enriched for NELL2 as compared to neurons and that NELL2 is robustly and significantly upregulated during oligodendrocyte maturation. Our data are in agreement with previous studies that showed A2B5^+^ cells isolated from adult human brain (Esmonde-White et al., 2019) and white matter tissues (Sim et al., 2006) exhibited significant enrichment of *NELL2* (fold change 2.37 and *p*-value 0.0004 as compared to A2B5^−^ cells). A2B5 is known to mark oligodendrocyte precursor cells (OPCs), and our data indeed demonstrate that NG2 and PDGRFA-expressing OPC progenitors express NELL2 and that NELL2 expression progressively increases during maturation into oligodendrocytes that express cnpase and O4 and subsequently MBP.

A comparison between our OL brain organoid data and published single-cell sequencing of early human fetal brain tissues not only confirms NELL2 is expressed in human oligodendroglial cells but also highlights differences. The quantification of oligodendroglial cells expressing *NELL2* in human *in vivo* fetal brain tissues revealed 30%–62% of oligodendroglial cells expressed NELL2 mRNA, whereas 69%–90% of oligodendroglial cells in human OL brain organoids expressed NELL2 protein. In addition to potential differences related to comparing NELL2 protein and RNA abundance, these differences may also be due to the fact that oligodendrogenesis in OL brain organoids is enforced *via* growth factor addition early during differentiation, whereas *in vivo*, this process is regulated by a variety of local factors and cues and occurs predominantly during post-natal development. Nevertheless, collectively, our data suggest that in humans, NELL2 may play a role in oligodendrocyte differentiation and function. In support of this notion, our AI analysis linked NELL2 to a range of diseases that are associated with abnormal myelination.

In summary, we found that human NELL2 is expressed in embryonic NSCs, OPCs, and neurons at varying levels but not in astrocytes. We further report for the first time that human oligodendrocytes express Nell2 and that this progressively increases during oligodendrocyte maturation. This finding, along with our transcriptome observations, indicates that human neural cells, excluding astrocytes, synthesize and express NELL2 protein during the peak stages of telencephalon neurogenesis and oligodendrogenesis and suggests that *NELL2* may be essential for proper cortical development. This hypothesis is further strengthened by the established role of Nell2 in guiding axonal growth and branching (Kim et al., 2020; Pak et al., 2020), a process fundamental to CNS development.

Our data reveal a number of roles of NELL2 in the CNS that are likely conserved between humans and other mammalian species and propose that in humans NELL2 has adopted additional roles in the oligodendrocyte lineage that may be linked to diseases that affect myelination.

In the future, it will be particularly interesting to carry out cell type-specific repression and overexpression studies of NELL2 in different cell types (such as rostral vs caudal NSCs) in human iPSC-derived brain organoid models to further elucidate the precise cell type-specific roles of cytosolic and secreted NELL2 isoforms during various stages of CNS development. Introduction of NELL2 domain-specific mutations and deletions may further enable mechanistic insights into the molecular mechanisms *via* which NELL2 performs its multitude of functions. The NELL2 expression data presented in this study should prove useful in enabling such further studies into the roles of NELL2 in human CNS development and its potential involvement in human white matter diseases.

## Materials and Methods

### Human Embryonic Stem Cell Culture and Generation of NSCs and Neurons

Human WTC iPSC (gift from Professor Bruce Conklin), G22 iPSCs, SOX10 mMaple iPSCs (Shaker M. R. et al., 2021), and H9-ESCs were cultured according to Stem Cell Technologies protocols feeder free in mTeSR (Stem Cell Technologies, Cat. #85851) medium on Matrigel (Stem Cell Technologies, Cat. #354277) (Shaker M. R. et al., 2021). To generate rNSCs, human iPSCs were treated with dual SMAD inhibitors 10 μM SB-431542 (Milteny Biotec, Cat. #130-106-543) and 0.1 µM LDN-193189 (Stemgent, Cat. #04-0074) as previously reported (Shaker M. R. et al., 2020). To generate cNSCs, human iPSCs were treated with SMAD inhibitor 10 μM SB-431542 (Milteny Biotec, Cat. #130-106-543) and 3 µM CHIR99021 (Sigma Aldrich, Cat. # SML1046), after which 0.1 µM retinoic acid (Sigma Aldrich, Cat. #R2625) was added on day 3. Both groups were cultured in N2 medium: DMEM/F12 (Gibco, Cat. #11320-33), 2% B-27 supplement (Gibco, Cat. # 17,504,044), 1% N-2 supplement (Gibco, Cat. #17502-048), 1% MEM Non-Essential Amino Acids (Gibco, Cat. #11140-050), 1% penicillin/streptomycin (Gibco, Cat. #15140148), and 0.1% β-mercaptoethanol (Gibco, Cat. #21985-023), for 7 days, and fresh medium was replaced daily. For neurosphere generation, induced NSCs were seeded onto ultra-low attachment culture dishes, and passaging was performed as described previously (Shaker et al., 2015). For neuronal generation, rNSCs were detached and dissociated into single cells using accutase (Gibco, Cat. #A11105-01). Dissociated rNSCs were seeded onto coverslips precoated with Poly-l-ornithine (Sigma, Cat. #P4957) and 5 μg/ml Laminin (Thermo Fisher, Cat. #23017015) on 18-mm glass cover slips in the presence of basic fibroblast growth factor (bFGF, 20 ng/ml; R&D, Cat. #233-FB-01M) for 12 h. Neurobasal medium (Gibco, Cat. #A35829-01) containing 2% B-27, 1% N-2, 1% penicillin/streptomycin, 0.025% insulin (Sigma, Cat. #I9278), 10 ng/ml BDNF (Lonza-Peprotech, Cat. #450-02-50), 0.2 μg/ml l-ascorbic acid (Sigma, Cat. # A4544), and 0.1 mM cAMP (Sigma, Cat. #D0627) was used to induce neuronal differentiation for 14 days.

### Generation of Brain Organoids

Brain organoids were generated with modifications as described previously (Lee et al., 2020b). Briefly, human iPSCs were treated for 3 days with dual SMAD inhibitors 10 μM SB-431542 (Milteny Biotec, Cat. #130-106-543) and 0.1 µM LDN-193189 (Stemgent, Cat. #04-0074) and cultured in N2 medium: DMEM/F12 (Gibco, Cat. #11320-33), 2% B-27 supplement (Gibco, Cat. # 17,504,044), 1% N-2 supplement (Gibco, Cat. #17502-048), 1% MEM Non-Essential Amino Acids (Gibco, Cat. #11140-050), 1% penicillin/streptomycin (Gibco, Cat. #15140148), and 0.1% β-mercaptoethanol (Gibco, Cat. #21985-023) to induce NEct cells. Sheets of NEct were gently detached with dispase and cultured using ultra-uncoated dishes in the presence of 60 ng/ml bFGF (R&D, Cat. #233-FB-01M) to allow the formation of neural spheroids (Supplementary Video S1). After 4 days of bFGF treatment, neural spheroids with a clear neuroepithelial edge were selected for Matrigel (StemCell Technologies, Cat. #354277) embedding. Embedded spheroids were then cultured over 13 weeks in terminal differentiation medium that consisted of the following: DMEM-F12 (Gibco, Cat. #11320-33): neurobasal medium (Gibco, Cat. #A35829-01), 0.5% N2 (Gibco, Cat. #17502-048), 12.5 µl of insulin (Sigma), 1% GlutaMAX, 1% MEM Non-Essential Amino Acids (Gibco, Cat. #11140-050), 1% penicillin/streptomycin (Gibco, Cat. #15140148), 0.1% β-mercaptoethanol (Gibco, Cat. #21985-023), and 1% B-27 supplement (Gibco, Cat. # 17504044).

OL brain organoids were generated as previously described (Shaker M. R. et al., 2021). Induced human NEct spheroids were cultured for 4 days with daily addition of 60 ng/ml bFGF (R&D, Cat. #233-FB-01M) in oligodendrocyte induction medium consisting of DMEM/F12 (Gibco, Cat. #11320-33), 2% B-27 minus vitamin A supplement (Gibco, Cat. # 17,504,044), 1% N-2 supplement (Gibco, Cat. #17502-048), 1% MEM Non-Essential Amino Acids (Gibco, Cat. #11140-050), 1% penicillin/streptomycin (Gibco, Cat. #15140148), 0.1% β-mercaptoethanol (Gibco, Cat. #21985-023), 10 ng/ml Human IGF-I (Lonza-PeproTech, 100-11-100), 10 ng/ml Human NT-3 (Peprotech, 450-03-50), 60 ng/ml 3,3′,5-triiodo-l-thyronine (Sapphire Bioscience, 000-23,845), 10 ng/ml HGF (Lonza-PeproTech, 100-39H), 100 ng/ml biotin (Sigma, B4639), 1 µM cAMP (Sigma, D0627), and 10 ng/ml PDGF-AA (Lonza-PeproTech, 100-13A). Upon embedding in Matrigel, these organoids were cultured in oligodendrocyte differentiation media consisting of DMEM-F12 (Gibco, Cat. #11320-33): neurobasal medium (Gibco, Cat. #A35829-01) 0.5% N2 (Gibco, Cat. #17502-048), 12.5 µl of insulin (Sigma), 1% GlutaMAX, 1% MEM Non-Essential Amino Acids (Gibco, Cat. #11140-050), 1% penicillin/streptomycin (Gibco, Cat. #15140148), 0.1% β-mercaptoethanol (Gibco, Cat. #21985-023), 1% B-27 minus vitamin A supplement (Gibco, Cat. # 17,504,044), 10 ng/ml Human IGF-I (Lonza-PeproTech, 100-11- 100), 10 ng/ml Human NT-3 (Peprotech, 450-03-50), 60 ng/ml 3,3ʹ,5-triiodo-l-thyronine (Sapphire Bioscience, 000-23,845), 10 ng/ml HGF (Lonza-PeproTech, 100-39H), 100 ng/ml biotin (Sigma, B4639), 1 µM cAMP (Sigma, D0627), and 10 ng/ml PDGF-AA (Lonza-PeproTech, 100-13A). Fresh medium was replaced three times a week.

### Generation of Human Spinal Cord Organoids

Human spinal cord organoids were generated as described previously (Lee et al., 2020b). Briefly, dissociated small clumps of hPSCs/hESCs were plated onto Matrigel-coated plates at high density in mTeSR1. After 2 days, mTeSR1 was replaced with differentiation medium (DM), consisting of DMEM/F-12 (Life Technologies, 11320033), 1% N2 (Life Technologies, 7502048), 2% B27 (Life Technologies, 17504044), 1% Non-Essential Amino Acids (NEAA) (Life Technologies, 11140050), 1% penicillin/streptomycin (P/S) (Life Technologies, 15,140,122), and 0.1% β-mercaptoethanol (Life Technologies, 21,985,023). To induce caudal neural stem cells, cells were treated with SB431542 (10 μM, TOCRIS, 1614) and CHIR99021 (3 μM, SIGMA, SML1046) in DM for 3 days with daily media change. On day 3, intact colonies were gently detached by 2.4 unit/ml dispase II (Life Technologies, 17105041) treatment for 20 min at 37°C. Detached colonies were then transferred onto uncoated culture dishes in DM supplemented with bFGF (20 ng/ml, R&D Systems, 233-FB). They began forming a neuroepithelial (NE) structure at the periphery surface of the organoid and were fed daily for 4 days. On day 7, organoids were cultured in DM containing retinoic acid (RA, 0.1 µM, SIGMA, R2625) without bFGF for 8 days. For maturation, organoids were grown in 1:1 mixture of DMEM/F-12 and neurobasal medium (Life Technologies, 21103-049; containing 0.5% N2, 1% B27, 0.5% NEAA, 1% P/S, 0.1% β-mercaptoethanol, 1% GlutaMAX (Life Technologies, 35,050-061), and 0.1 µM RA. The medium was changed every 3–5 days.

### Quantitative Real-Time Polymerase Chain Reaction

Total RNA was isolated from organoids as described previously (Shaker M. R. et al., 2020) using TRIzol (Invitrogen, 15596026) in triplicate according to the manufacturer’s instructions. A total of 1 µg of RNA was utilized to generate cDNA using the First-Strand cDNA Synthesis Kit (Thermo Scientific, Cat. #K1612), and SYBR Green (Applied Biosystem, Cat. #A25742) was used for qPCR. All experiments were performed in biological quadruplicate or more for each sample analyzed. GAPDH was used to normalized expression values. Primers were designed using the free online system (NCBI), and primers are listed in Supplementary Table S3. GraphPad Prism 8.3.1^®^ software and Sigma Plot 13.0^®^ software were utilized to calculate means and standard deviations.

### Histology and Immunostaining Analysis

Tissues of brain organoid were processed as described before (Lee et al., 2019). Tissues were fixed with 4% paraformaldehyde (PFA) (Thermo Fisher, Cat. #ALF043368.9M) for 60 min at room temperature (RT) before washing three times with 1X PBS for 10 min at RT. Washed organoids were then immersed in 30% sucrose at 4°C until organoids were sunk, before embedding in a solution made of 3:2 OCT and 30% sucrose on dry ice. Immunohistochemistry (IHC) was performed as described before (Kim et al., 2017). Sectioned organoids were washed two times with 1X PBS to remove the excess of OCT before blocking for 1 h with a blocking solution made of 3% bovine serum albumin (Sigma, Cat. A9418-50G) and 0.1% Triton X-100 in 1X PBS. Diluted primary antibodies in blocking solution were added overnight at 4°C before adding appropriate secondary antibodies, mounting, and imaging. The immunocytochemistry (ICC) was performed as described before (Shaker et al., 2015). NSCs and neurons were fixed with 4% PFA in 1X PBS for 10 min at RT before washing twice with 1X PBS for 10 min at RT. Primary and secondary antibodies were added as described above. All tissues and cells were counterstained with Hoechst 33342 (Invitrogen, Cat. #H3570), and images were captured using confocal microscopy (Leica TCS SP8) based in the SBMS Imaging Facilities of the University of Queensland. The primary antibodies used in this study are listed in Supplementary Table S4.

### Single-Cell Ribonucleic Acid Sequencing

The hSCOs were collected in a Petri dish on day 100 and chopped into small pieces. After dissection, the hSCOs were dissociated by using papain containing l-cysteine by incubation at 37°C with gentle shaking. Papain was removed after 30 min, and the dissociated cells were washed twice with ice-cold HBSS. Libraries were prepared using the Chromium Controller according to the 10x Single Cell 3′ v3 protocol (10x Genomics). Briefly, the dissociated cell suspensions were diluted in nuclease-free water to achieve a targeted cell count of 10,000. The cell suspension was mixed with a master mix and loaded with Single-Cell 3′ Gel Beads and Partitioning Oil into a Single Cell 3′ Chip. RNA transcripts from single cells were uniquely barcoded and reverse-transcribed within droplets. cDNA molecules were pooled. The cDNA pool was subjected to an end repair process, followed by the addition of a single “A” base and ligation of the adapters. The products are then purified and enriched with PCR to develop the final cDNA library. The purified libraries were quantified using qPCR based on the qPCR Quantification Protocol Guide (KAPA) and qualified using the Agilent Technologies 4200 TapeStation (Agilent Technologies). The libraries were subsequently sequenced using the HiSeq platform (Illumina) with a read length of 28 bp for read 1 (cell barcode and unique molecular identifier, UMI), 8 bp index read (sample barcode), and 91 bp for read 2 (RNA read). The preprocessing and analysis of single-cell RNA-seq data were performed as described before (Lee et al., 2020b).

### Bioinformatics Analysis

Normalized data of microarray GSE132089 (Shaker M. R. et al., 2020) was downloaded from GEO of the NCBI free online system. cNSCs was used a control group to calculate the fold change and used to identify significantly enriched genes in rNSCs based on adjusted *p*-value. The significantly enriched genes were utilized to generate Gene Ontology enrichments (http://bioinformatics.sdstate.edu/go/) associated with cellular components, molecular function, KEGG, and biological process. The GeneMANIA plugin of the Cytoscape free software was employed to identify hub genes based on the interaction with other genes. The normalized data of RNA-seq E-MTAB-2268 (Gouti et al., 2014) was downloaded from the Array Express database (http://www.ebi.ac.uk/arrayexpress). The Ingenuity^®^ Pathway Analysis (IPA^®^) software was applied to generate a list of genes significantly enriched in N_H_ NSCs compared to N_P_ NSCs. Venn diagram was generated using the free online tool (http://bioinformatics.psb.ugent.be/webtools/Venn/). Gene expression analysis of scRNA-seq data was performed using Seurat v4.0 (Hao et al., 2021) running on R v4.1.2 with R-studio v3.0.1. Briefly, read count data and associated metadate were combined to build a Seurat object; then, cells that have less than 200 unique feature counts and more than 5% mitochondrial gene counts were removed from the dataset. The expression data was log-normalized and scaled, followed by JackStraw dimensionality analysis (Macosko et al., 2015), cluster identification, non-linear dimensionality reduction, and identification of differentially expressed genes. The normalized and clustered data of analyzed single cells were accessed using the online tSNE browser (bit.ly/cortexSingleCell) and include an interactive plot of the data with cluster designations (Nowakowski et al., 2017).

### Magnetic Activated Cell Sorting

Dissociation of brain organoids (*n* = 5) was carried out by incubation in accutase (StemPro) under 80 rpm orbital agitation at 37°C for 20 min. Remaining clumps were mechanically dissociated with a 1-ml pipetting tip, and the homogenate was passed through a cell strainer to isolate only single cells that were subsequently processed for MACS cell sorting according to the manufacturer protocol. Briefly, dissociated cells were incubated with microbeads conjugated to O4 antibody (Miltenyi #30-096-670) for 15 min a 4°C. After washing, the O4-positive cells were isolated by passing through the LS MACS magnetic column (Miltenyi #130-042-401). The eluted cells were then similarly processed by sequential incubation with a biotinylated anti-GLAST antibody and anti-biotin microbeads (Miltenyi #130-095-825), followed by elution through another LS MACS magnetic column. The final fraction, deprived of O4 and GLAST-positive cells, was considered the remaining brain organoid cell fraction. Sorted cells were then processed for western blotting.

### Western Blot

Western blotting was performed as previously described (Kim et al., 2017). Briefly, neurospheres and sorted single cells were collected and lysed using a sonicator in Pierce^TM^ RIPA buffer (Thermo Fisher Scientific, Cat. #89900) containing a cocktail of protease and phosphatase inhibitors (Roche). The concentration of proteins was then calculated using Pierce^TM^ bicinchoninic acid (BCA) protein assay kit (Thermo Fisher Scientific, Cat. #23227) following the manufacturer’s instructions. All proteins were adjusted to 1 μg/μl concentration followed by heating for 10 min at 100°C before loading. Then, 30 µg of proteins was loaded and separated on Mini-PROTEAN TGX Stain-Free Gels (Bio-Rad, Cat. #4568044). Following separation, the proteins were next transferred onto iBlot 2 PVDF Mini Stacks (Invitrogen, Cat. #IB24002). To block the membrane for 1 h at RT, 5% skim milk in TBST (20 mM Tris–HCl, pH 7.6, 136 mM NaCl, and 0.1% Tween-20) was used, before adding the primary antibody. The primary antibody was diluted in 5% BSA for 12 h at 4°C. The primary antibodies are listed in Supplementary Table S4. The membrane was then washed three times with 1× TBST for 10 min each at RT followed by incubation with host corresponding secondary antibody diluted at 1:5,000 in 5% skim milk made with 1× TBST for 1 h at RT. The membrane was again washed three times with 1× TBST for 10 min each at RT before visualization with Clarity Western ECL Substrate (Bio-Rad, Cat. #170-5060).

### Target–Disease Association Prediction Platform Based on Artificial Intelligence

In this study, we used the Standigm ASK platform (https://icluenask.standigm.com) to identify predictions of NELL2-related diseases. The Standigm ASK platform is a web-based platform that predicts the target–disease relationship using an AI-based knowledge graph. In the platform, score is the prioritization value given by the algorithm between 0 and 1. Novelty field shows values denoting the statistical significance of co-occurrence of the specific disease–target pair. Larger values indicate a more unexpected association. The Standigm ASK platform predicted 10,996 human diseases associated with NELL2. Only CNS-related diseases were extracted by applying additional filtering keywords including central nervous system, neuro, and myelin. The top 10% of predicted human NELL2-related diseases are listed in Supplementary Table S2.

### Image and Statistical Analysis

All data are presented as the mean ± standard deviation of the mean of at least three independent experiments. For the length of NELL2 puncta measurements, cells were imaged using the confocal microscopy (Leica TCS SP8). The LAS X software was used to generate the scale bar corresponding to the objective lens. Images of stained cells were exported to ImageJ to measure the diameter of individual NELL2 puncta. The pixel length of the longest distance was measured and assigned as the length of NELL2 puncta and calibrated against a given scale. The number of biological replicates as well as the sample size are indicated in the figure legends, and power analysis was used to calculate the sample size. Student’s *t*-test was employed to compare two groups, and one-way ANOVA was applied for comparing multiple groups, followed by the Tukey’s *post*-*hoc* analysis for comparisons to a single control. Statistical analysis was performed using Sigma Plot 13^®^ software. The *p*-value of <0.05 was defined as a measure of minimal statistical significance.

## Data Availability

The data that support the findings of this study are available from the corresponding authors upon reasonable request. The datasets presented in this study can be found in online repositories. The names of the repository/repositories and accession number(s) can be found at NCBI with accession GSE190633.
