# Supplementary figures and images for: Neural Epidermal Growth Factor-Like Like Protein 2 Is Expressed in Human Oligodendroglial Cell Types
Source: Front Cell Dev Biol. 2022 Feb 21;10:803061. doi: 10.3389/fcell.2022.803061 (PMC8899196; doi:10.3389/fcell.2022.803061)

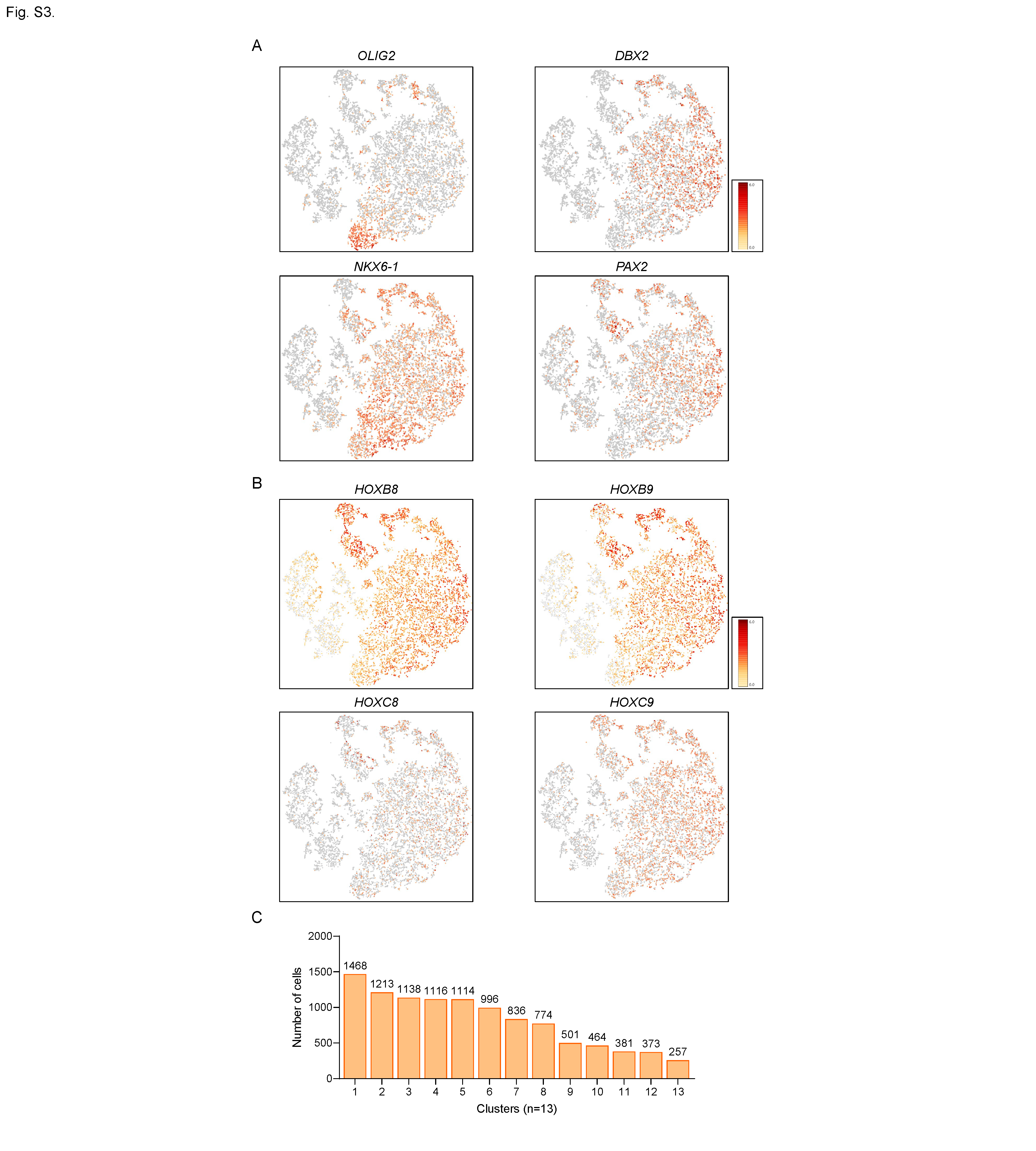

Supplement: Supplementary file 1 [file Image3.tiff]

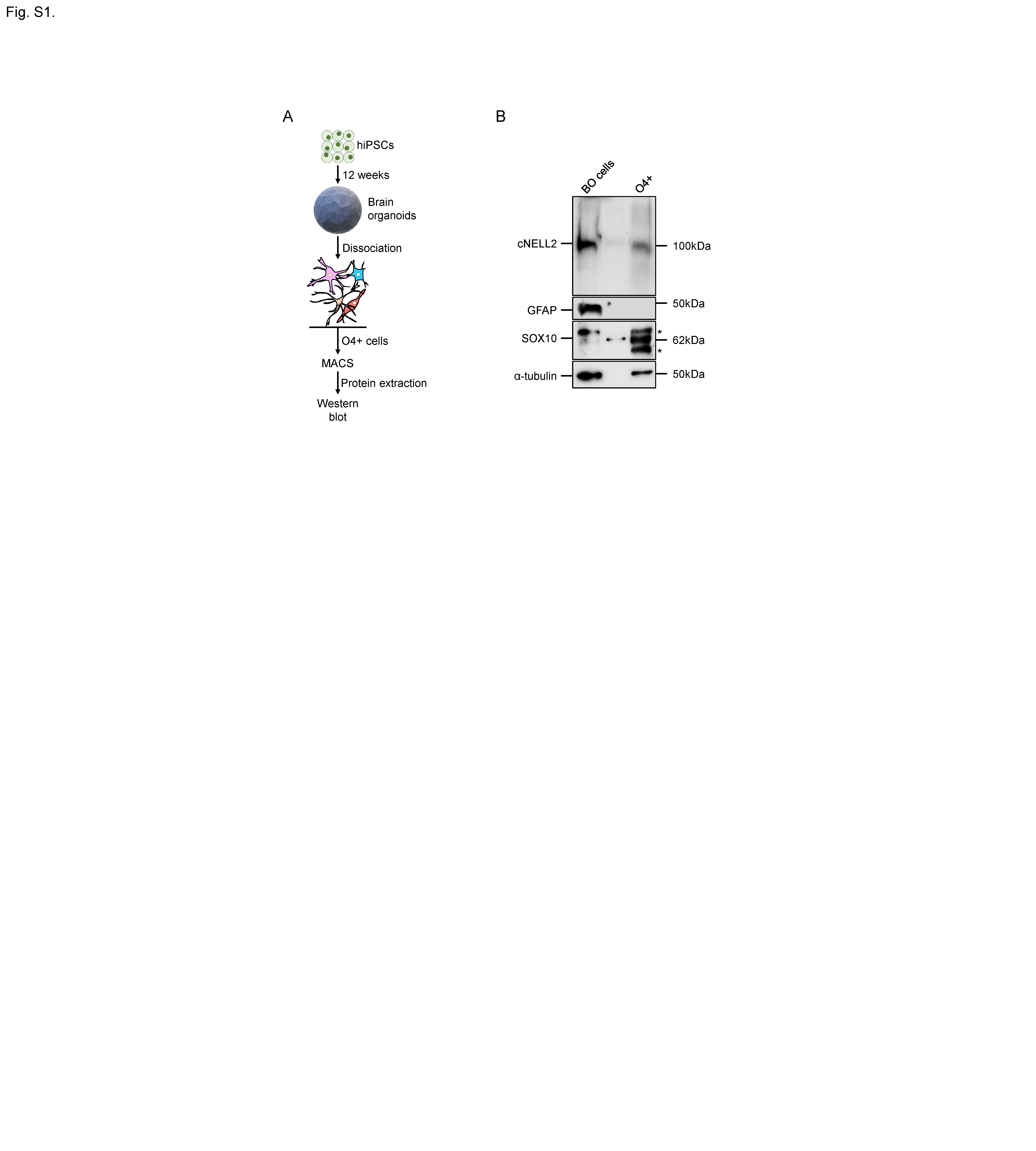

Supplement: Supplementary file 2 [file Image1.tiff]

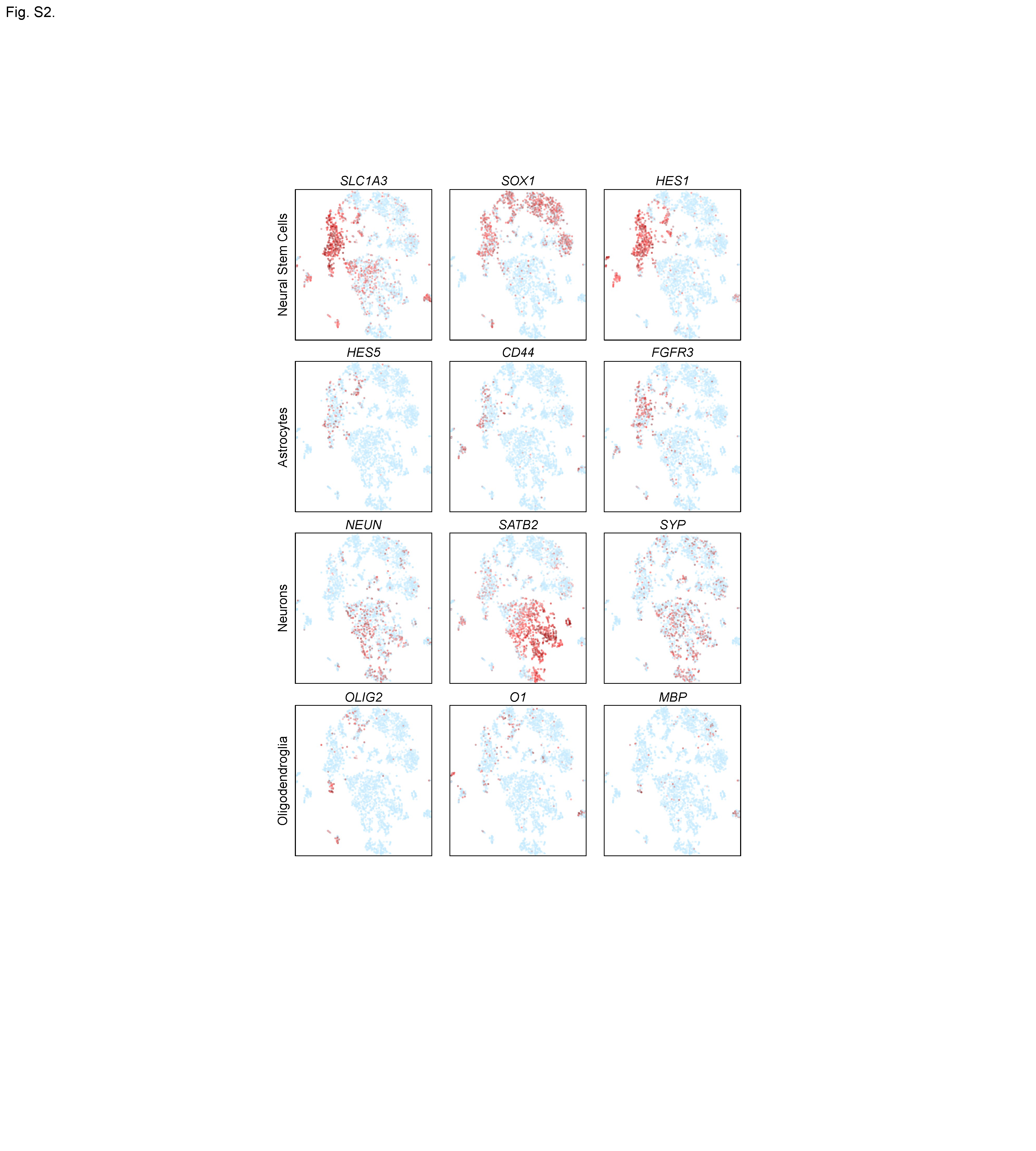

Supplement: Supplementary file 3 [file Image2.tiff]
